# Maternal stress is associated with higher protein-bound amino acid concentrations in human milk

**DOI:** 10.3389/fnut.2023.1165764

**Published:** 2023-09-07

**Authors:** Hannah G. Juncker, Eva F. G. Naninck, Britt J. van Keulen, Jolinda E. Harinck, Lidewij Schipper, Paul J. Lucassen, Johannes B. van Goudoever, Susanne R. de Rooij, Aniko Korosi

**Affiliations:** ^1^Brain Plasticity Group, Swammerdam Institute for Life Sciences, University of Amsterdam, Amsterdam, Netherlands; ^2^Emma Children's Hospital, Amsterdam University Medical Centers, University of Amsterdam, Vrije Universiteit, Amsterdam, Netherlands; ^3^Amsterdam Reproduction and Development, Amsterdam, Netherlands; ^4^Danone Nutricia Research, Utrecht, Netherlands; ^5^Department of Epidemiology and Data Science, Amsterdam University Medical Centers, University of Amsterdam, Amsterdam, Netherlands; ^6^Amsterdam Public Health Research Institute, Aging and Later Life, Health Behaviors and Chronic Diseases, Amsterdam, Netherlands

**Keywords:** breast milk, lactation, postpartum stress, amino acid, early life stress

## Abstract

**Background:**

Maternal stress in the postpartum period affects not only the mother but also her newborn child, who is at increased risk of developing metabolic and mental disorders later in life. The mechanisms by which stress is transmitted to the infant are not yet fully understood. Human milk (HM) is a potential candidate as maternal stress affects various components of HM, e.g., fat and immunoglobulin concentrations. To date, it is unknown whether maternal stress also affects the amino acids (AAs) in HM, even though this nutrient is of extreme importance to child health and development. This study aimed to investigate whether and how maternal stress is associated with the AA composition of HM.

**Methods:**

In this observational cohort study (Amsterdam, The Netherlands), lactating women were recruited in two study groups: a high-stress (HS) group; women whose child was hospitalized (*n* = 24), and a control (CTL) group; women who gave birth to a healthy child (*n* = 73). HM was collected three times a day, on postpartum days 10, 17, and 24. Perceived psychological stress was measured using validated questionnaires, while biological stress measures were based on hair, saliva, and HM cortisol concentrations. HM protein-bound and free AAs were analyzed by liquid chromatography and compared between groups.

**Results:**

Maternal perceived stress scores were higher in the HS group (*p* < 0.01). The concentrations of protein-bound AAs in HM were higher in the HS group compared to the CTL group (*p* = 0.028) and were positively associated with HM cortisol concentrations (*p* = 0.024). The concentrations of free AAs did not differ between study groups and were unrelated to cortisol concentrations.

**Conclusion:**

Findings from this prospective cohort study suggest that maternal stress in the postpartum period is associated with an altered human milk amino acid composition, which could play a role in the transmission of maternal stress effects to her child. The physiological implications of these stress-induced changes for infant development await future research.

## 1. Introduction

Maternal stress in the postpartum period not only affects the mother but may also have consequences for her newborn child. It has been shown that maternal stress occurring during this sensitive developmental period is associated with the infant's risk of developing a wide range of disorders, including metabolic and mental health disorders (1–3). Because the prevention of stressful maternal circumstances in the early postnatal period is generally difficult, a better understanding of the processes underlying these detrimental consequences for the infant is needed. Several mechanisms by which transmission of maternal stress to her infant occurs have been suggested, one of them being stress-induced changes in human milk (HM) composition (4, 5).

HM is the optimal source of nutrition for newborn infants (6–8). It is a highly complex fluid, consisting of over 100 different components that are influenced by many different factors (9). It has been demonstrated previously that maternal psychopathology and maternal stress in the postpartum period are associated with an altered composition of fatty acids in HM (10–16). However, whether maternal stress is also associated with changes in other HM nutrients, e.g., amino acids (AAs), is so far unknown. As AAs in HM are critical for infant growth and development and are necessary for almost all infant body processes (17), it is important to understand how they are affected by maternal stress. In addition, previous human studies point toward an effect of stress on AAs in the plasma, and previous animal experimental studies even suggest a change in milk composition under the influence of stress.

In both human and animal plasma, AAs decrease as a result of stress (18–20). As maternal plasma is the source from which AAs are transported into milk, lower maternal plasma AA levels, e.g., due to stress, may likely result in lower AA levels in milk (21). Whereas, human studies addressing this aspect are lacking, two studies in mice have shown that while maternal stress resulted in lower concentrations of AA in maternal plasma and reduced growth of the offspring, the concentrations of the AAs asparagine and alanine were increased in milk (22, 23). In another animal study, maternal stress during lactation lowered methionine levels in the offspring brain and plasma and induced cognitive deficits later in life, which, notably, could be partly counteracted by methionine supplementation in the dam's diet (24). While this suggests that maternal stress lowers methionine levels in milk, which could have important programming effects on brain health, the AA concentrations in milk were not determined in this study (24).

This study aimed to investigate whether maternal stress in the first month postpartum is associated with the fraction of protein-bound AA (BAA), which makes up 90–95% of the AAs, and the free AA (FAA), which comprises a relatively small fraction of the AA in HM (17). We further focused on methionine as a key AA that may play a role in the long-term consequences of early life stress and investigated the associations between maternal stress and this specific AA. A better understanding of potential stress-induced changes in HM AA composition will contribute to our knowledge of how maternal stress can be transferred to the infant.

## 2. Materials and methods

### 2.1. Research design and study population

We studied a prospective observational cohort of lactating women who were followed over their first month postpartum, and experienced various amounts of stress. Participants were recruited during pregnancy or within the first 10 days after giving birth, via social media, flyers at midwife practices, or at the maternal or neonatal ward of the Amsterdam University Medical Center (Amsterdam, The Netherlands). Mothers were eligible to participate when they were 18 years of age or older, and if they had the intention to breastfeed their infant for at least the first month after birth. Exclusion criteria were as follows: (1) maternal (gestational) diabetes mellitus, as the glucocorticoid system may be regulated differently (25, 26), (2) maternal use of psychopharmaceuticals or glucocorticoid medication, as this may interfere with questionnaire scoring, glucocorticoid system regulation, and maternal cortisol concentrations (27), and (3) major congenital disease of the neonate and/or a life expectancy of the neonate of <1 month (duration of the study).

To ensure the inclusion of a large enough range of stress levels among the included participants, two groups of women who delivered at term were included: a high-stress group (HS group) and a control group (CTL group). Women were included in the CTL group when they gave birth to a healthy infant at term. Women were included in the HS group when they gave birth to an infant at term who was admitted to the hospital for a minimum of 2 days. Hospitalization of the infant was considered a maternal stressor.

Recruitment took place in The Netherlands between November 2017 and December 2019. Written informed consent was obtained from all participants prior to participation. This study was approved by the Ethics Committee of the Amsterdam University Medical Centre, AMC on 2 May 2017 (METC 2017025, NL59994.018.16) and conducted in accordance with the Declaration of Helsinki.

### 2.2. Data collection and storage

#### 2.2.1. Study timeline

**Figure 1:**
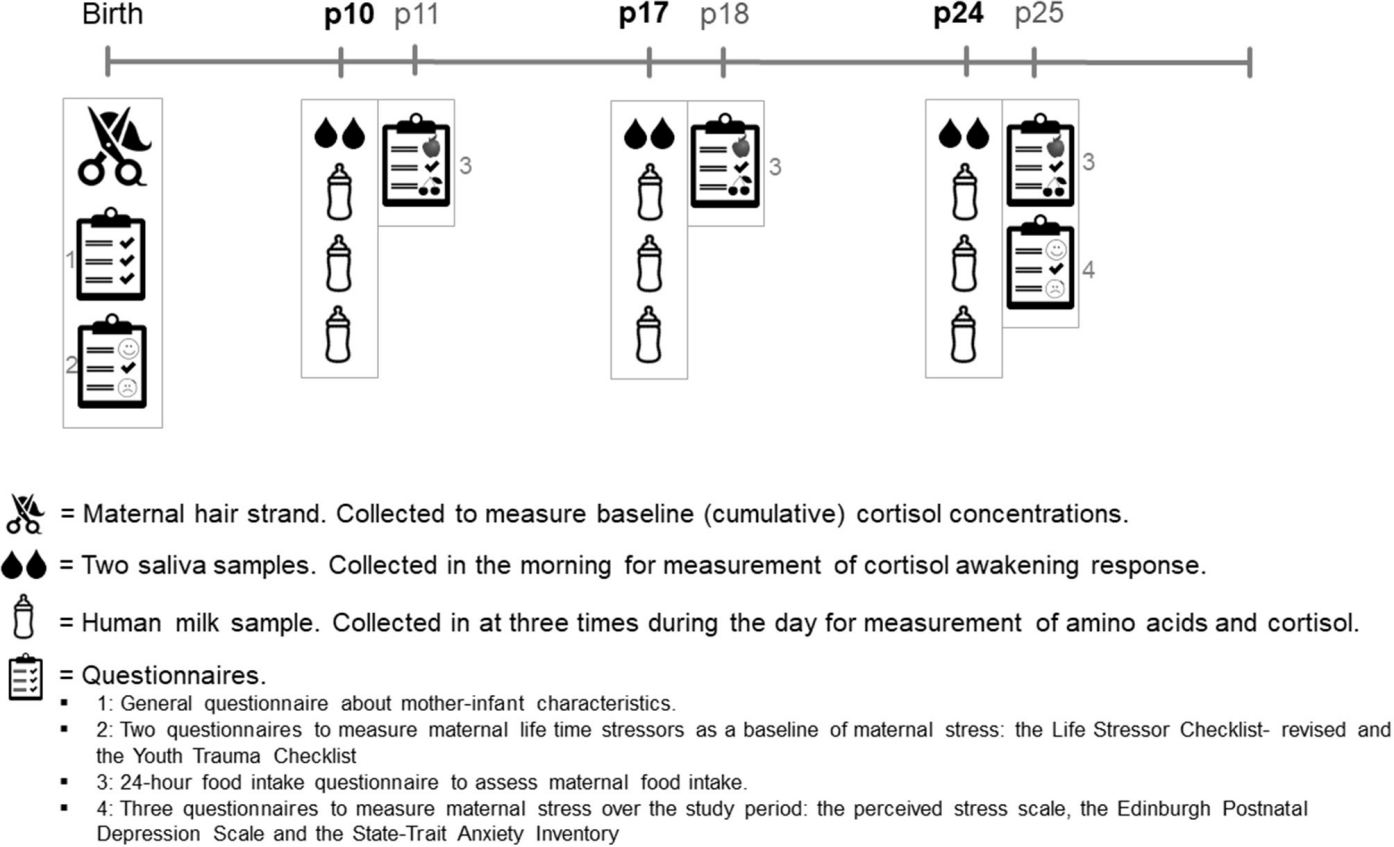
Timeline of study procedures. *p*, postpartum day.

Figure 1 shows the study timeline. Recruitment took place within the first 10 days postpartum. After the participant's recruitment, a strand of hair was collected for glucocorticoid measurements, and the participants completed a questionnaire about their general health, their pregnancy, and their lifetime stress experiences. The study collected data on 3 collection days: at postpartum (P) days 10, 17, and 24. On these collection days, women collected two saliva samples and three HM samples. After each collection day, the participants filled out a 24-h food recall questionnaire. At the end of the study, participants filled out three questionnaires about their stress experiences during the study period. See the items below for further details on sample collection, measurements, and questionnaires.

#### 2.2.2. Hair sample collection and storage

A strand of hair (~100 hairs, 3 mm diameter) was cut by the researcher as close to the scalp as possible at the posterior vertex position. The hair was stored in the dark at room temperature until analysis. A short questionnaire was filled out by the participants in order to correct for factors influencing hair glucocorticoid concentrations. Hair samples were analyzed for cortisol and cortisone as a baseline biological stress measurement reflecting the last trimester of pregnancy (28, 29).

#### 2.2.3. Saliva sample collection and storage

Saliva was collected two times in the morning on every collection day to measure the cortisol awakening response. Participants were instructed to chew on a swab (Salivette, Sarstedt, Nümbrecht, Germany) for 1 min. The first sample (S1) was obtained within 0–10 min after waking, and the second sample (S2) was obtained 30–45 min after waking (30). Participants were requested to write down their wake-up time and the time of saliva collection. After collection, saliva samples were sent to the study site, where they were centrifuged, aliquoted, and stored at −20°C until analysis.

#### 2.2.4. Milk sample collection and storage

On every collection day (P10, P17, and P24), participants collected three HM samples in which concentrations of AAs and cortisol/cortisone were measured. HM cortisol/cortisone was measured to be able to directly correlate this with the HM AAs. To take into consideration, the circadian rhythm of HM cortisol and to make sure that circadian variation in HM AAs was represented in the samples (31, 32), participants were instructed to collect one HM sample in the morning, one in the afternoon, and one in the evening on each day that they collected milk. To make sure the HM sample would contain a mixture of both foremilk and hindmilk, participants were requested to fully empty one breast before feeding their infant, mix the milk, and thereafter put 5 ml of HM in a sterile polypropylene container (Sarstedt, Germany) for analysis. Participants were free to choose from which breast the milk was collected. Participants were asked to record the date and time of milk collection, the pumping method used (i.e., manually or with an electric pump), and the total amount of milk collected. Participants stored the milk samples in their freezer (−20°C) up until collection by the researcher. At the study site, HM samples were stored at −20°C until analysis.

### 2.3. Questionnaires

The questionnaires used in the study are described below. For the analyses, the total questionnaire scores and their ranges were used. To establish the participant's lifetime stress exposure, the participants filled out two questionnaires at the start of the study.

#### 2.3.1. The life stressor checklist-revised

The Dutch version of the LSC-r questionnaire. This checklist is a 26-item scale to identify exposure to traumatic events or other stressful life events (33). Each item questions whether a certain event happened in the participant's life (34).

#### 2.3.2. The youth trauma checklist

The Dutch version of the JTV questionnaire (25 items) is a self-reported inventory that provides a brief and relatively non-invasive retrospective assessment of early life traumatic experiences (35). The JTV discriminates against five domains of abuse/neglect (physical, sexual and emotional abuse, and physical and emotional neglect).

#### 2.3.3. Twenty-four-hour food recall questionnaire

After each collection day, participants received a 24 h-recall using the digital program Compl-eat™ developed by the Department of Human Nutrition at Wageningen University (the Netherlands) (36). This questionnaire assesses the exact food intake (in grams per day) on the collection day. The Compl-eat™ web-based module was specifically designed for the Dutch population and guides participants to accurately report all foods and drinks consumed during the previous 24 h. Information on AA supplement intake was taken into account in the 24 h-recall. The mean intake for protein and all AAs of the three collection days was calculated and used as a measure of maternal intake over the study period.

A measure of psychological stress levels *during* the study period was obtained by three questionnaires that the participants filled out at the end of the study, concerning the levels of stress they experienced during the past month.

#### 2.3.4. Perceived stress scale

The perceived stress scale is a validated 14-item questionnaire. The questionnaire determines the degree to which certain situations are experienced as stressful (37, 38). Each question is scored on a 5-point Likert scale.

#### 2.3.5. Edinburgh postnatal depression scale

The Dutch version of the well-validated EPDS is a 10-item self-inventory to assess symptoms of depression and/or anxiety in women who recently gave birth (39).

#### 2.3.6. State-trait anxiety inventory

The Dutch version of the STAI is a well-established measure of trait and state anxiety and consists of two parts. The first part of this inventory, the STAI-state (STAI-s), contains 20 items to assess anxiety at this moment, rated on a 4-point intensity scale. The second part, the STAI-trait (STAI-t), contains 20 items and assesses anxiety, in general, rated on a 4-point intensity scale (40).

### 2.4. Laboratory analysis

#### 2.4.1. Hair cortisol/cortisone

For analyses, the proximal 3-cm hair segment was used. Wash and steroid extraction procedures were performed as described by Stalder et al., with some changes being made to allow analysis by liquid chromatography-tandem mass spectrometry (LC-MS/MS) (41). The lower limits of quantification were below 0.1 pg/mg for cortisol and cortisone. The inter- and intra-assay coefficients of variance were between 3.7 and 8.8%.

#### 2.4.2. Saliva cortisol/cortisone

Cortisol and cortisone in saliva were determined using supported liquid extraction (SLE+) followed by LC-MS/MS detection. Quantification was performed using an isotope dilution, with the limit of quantification being 0.3 nmol/L. The mean intra-assay variation was 6 and 7% for cortisol and cortisone, respectively.

#### 2.4.3. HM cortisol/cortisone

For each HM sample, cortisol and cortisone were determined using liquid extraction followed by SLE+ and LC-MS/MS detective as described earlier by van der Voorn et al. (42). Quantification was performed using an isotope dilution.

#### 2.4.4. Protein-bound AA in HM

For the determination of BAA in HM samples, the three milk samples from 1 collection day were mixed to have a good representation of BAA concentrations during the whole day. Thereafter, 0.20 ml of diluted hydrochloric acid containing 0.5% 2-mercaptoethenol was added to the HM sample and mixed. The present oxygen was removed by flushing the headspace of the tube with nitrogen for 60 s. The protein was hydrolyzed by heating the mixture for 20–22 h. When the mixture was cooled down, 0.20 ml of sodium hydroxide solution, 2.0 ml of demi water, and 0.2 ml of internal standard (Norvaline 20 μg) were added and mixed. The mixture was centrifuged, and a small part of the liquid was filtered over a 0.45 mm polyvinylidene difluoride filter. The peak area of each AA was calculated using the LabSolutions software from Shimadzu and compared to the peak area of the internal standard (Sigma).

#### 2.4.5. Free AA in HM

The mixed HM samples were also used to determine the free AA in HM. An ultra-fast liquid chromatography (UFLC)-based protocol was used. Each 50 μl of milk sample was mixed with 1.0 ml of internal standard solution (2.5 mg/ml of L-norvaline). This mixture was centrifuged, and 25 μl of supernatant was transferred into a sample vial. A pre-column derivatization process was carried out by adding 30 μl of o-phthalaldehyde (OPA) reagent to the vial and mixing three times with a mixing volume of 45 μl. One microliter of this OPA-derivatized sample was injected and analyzed in a UFLC system with fluorimetry to detect the signal.

Standard AA solution Sigma AA-S-18 was used for calibration. To prepare the calibration AA solution, asparagine, and tryptophan were added in to Sigma AA-S-18 stock solution to reach a concentration of 2.5 μM/ml of each AA. Next, 0.50, 1.0, 2.0, and 5.0 ml of this solution were mixed with 1.6 ml of perchloric acid and further diluted to 50 ml. The calibration AA solution was prepared to OPA derivate as described previously and measured in an UFLC system. The calibration curve was constructed from peak areas and AA concentrations. Response factors for each AA were obtained by an extra analysis of a standard AA solution containing internal standards.

### 2.5. Statistical analysis

Sample characteristics were described as mean with standard deviation (SD), median with 25th and 75th percentiles (Q1–Q3), or frequencies. To test differences in maternal and infant characteristics (including maternal BMI and infant sex), dietary AA intake over the study period, and stress measurements (questionnaires and cortisol) between both study groups, unpaired Student's *t*-tests (for continuous normally distributed data), chi-square tests (for binary categorical data), Mann–Whitney *U*-tests (for continuous not normally distributed data), or linear mixed models (for data that were measured at multiple time points) were used. The mean of the three 24-h recalls was used to compare the dietary intake of AA between groups, as this reflects the overall intake of the participants during the study period.

The HM cortisol area under the curve (AUC) was calculated to provide a value that better reflects HM cortisol throughout the day, which is known to follow a circadian rhythm (31). Therefore, all HM cortisol values were standardized to 7:00 a.m., 14:00, and 22:00 by regressing the time of collection to cortisol values. For each participant, we then calculated the estimated cortisol value at 07:00, 14:00, and 22:00. To do this, the following formula was used: HM cortisol in mmol/L ± unstandardized regression coefficient of all HM cortisol values ^*^ [new (standardized)] time point – real-time point) (43). Subsequently, the HM cortisol AUC for each collection day was calculated as described by Pruessner et al. (44). The cortisol value at 7:00 a.m. was considered the HM cortisol morning peak (31). The highest cortisol value of the two morning saliva samples was considered the saliva cortisol morning peak. When the time of S1 collection was >30 min after waking up, saliva values were excluded.

Analyses were performed separately for BAA and FAA. Before analysis, the HM AAs were categorized into different outcome variables: total AA, essential AA, and non-essential AA. Because AA from one precursor family can be converted into other members of this family, AAs were also grouped into precursor groups: the glutamate precursor group (glutamic acid, glutamine, and arginine), the aspartate precursor group (aspartic acid, methionine, isoleucine, threonine, and lysine), the serine precursor group (serine, glycine), the pyruvate precursor group (valine, leucine, and alanine), the aromatic precursor group (phenylalanine, tyrosine, and tryptophan), and the histidine precursor group (histidine). Methionine was analyzed separately (24).

All outcome variables were checked to see whether they were normally distributed. When variables were not distributed normally, a log transformation was performed. When participants completed <1 full day of sample collection, they were excluded from the final analysis.

To answer the research question whether and how maternal stress affects the BAA and FAA concentrations in HM, the AA outcomes as described above were compared between the HS and CTL groups. As all study time points were taken into account in the comparison, linear mixed models were used to analyze the group differences to control for within-person repeated measures. The analysis was corrected for factors differing between study groups. In addition, we tested whether maternal dietary AA intake during the study period differed between study groups. If the maternal dietary intake of a specific AA statistically differed between groups, the comparison of that specific HM AA was corrected for the maternal intake. As HM AA concentrations differ between the different weeks and stages of lactation, the interaction between the study time point and study group was investigated and reported.

To answer the research question of whether HM cortisol concentrations (cortisol AUCs) are related to HM AA concentrations, we performed a secondary analysis. The relationship between HM AA and HM cortisol AUCs was investigated independently of the study group. This relationship was only investigated for total AA, essential AA, non-essential AA, and methionine in HM. As all study time points were taken along in this secondary analysis, linear mixed models were used to control for within-person repeated measures. The analysis was corrected for potential confounding factors that have been shown to influence the AA composition of HM in the previous literature: AA dietary intake and maternal BMI (17, 45–47). Due to the explorative nature of the study, the statistical analyses were not corrected for multiple testing. To reduce the number of statistical tests and the likelihood of a type 1 error, most of the separate AAs were categorized into their precursor families and analyzed as such. Statistical analyses were two-sided. A *p*-value of <0.05 was considered statistically significant. Statistical analyses were performed using IBM SPSS Statistics for Windows, version 27. GraphPad Prism 9 for Windows was used to display the results.

## 3. Results

### 3.1. Maternal characteristics, food intake, and stress measures

**Figure 2:**
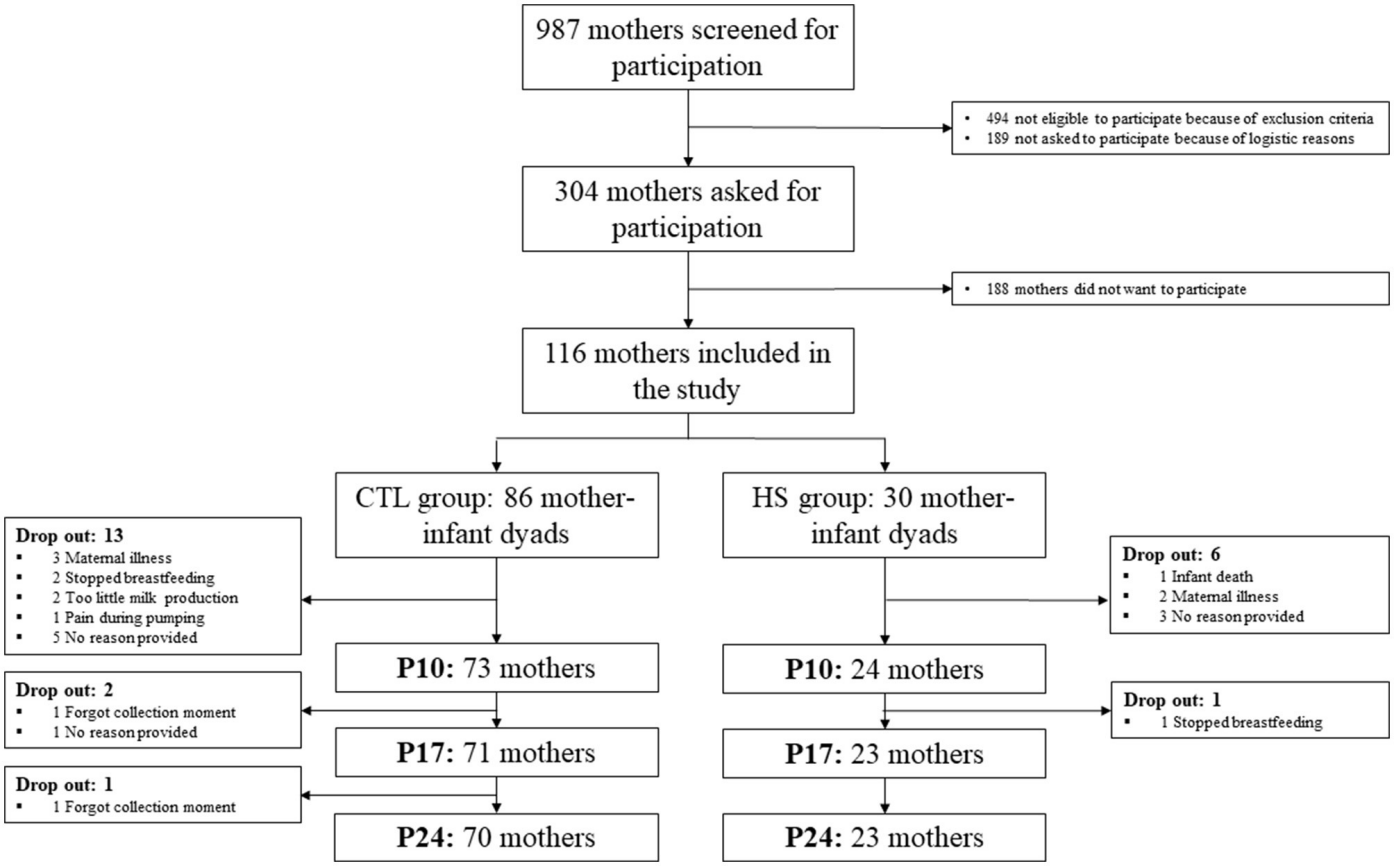
Flowchart of the study population. CTL group, control group; HS group, high-stress group; p, postpartum day.

In total, 86 lactating women were included in the CTL group and 30 in the HS group. Nineteen women stopped participating before the end of the study due to various reasons (see Figure 2 for drop-out reasons), 13 women (15%) in the CTL group, and six women (20%) in the HS group (Figure 2). Characteristics and dietary protein and AA intake of the participants are shown in Tables 1, 2, respectively. Maternal baseline characteristics, including maternal age, BMI, ethnicity, education level, alcohol consumption, smoking, dietary habits, and mode of delivery, did not differ between the study groups. Maternal protein/AA intake during the study period was also similar between both study groups. In addition, the HM pumping method used and the storage time of the samples did not differ between study groups. The only difference between study groups was that mothers in the HS group gave birth to a male infant more often than mothers in the CTL group, 75 and 49%, respectively (*p* = 0.048); the birthweights did not differ between study groups. In the HS group, hospitalization duration ranged between 2 and 12 days, with a median of 7 days. The duration of hospitalization in this group was not associated with maternal stress scores (*p* = 0.282 for PSS score).

**Table 1:** Maternal and infant baseline characteristics.

	**CTL (*n* = 75)**	**HS (*n* = 24)**	***p*-value**
**Maternal characteristics**			
Age, mean (SD)^a^	32.4 (3.3)	32.5 (3.6)	0.815
BMI (kg/m^2^), mean (SD)^a^	23.1 (3.7)	24.3 (5.5)	0.246
Ethnicity %^b^			0.338
Dutch	88.0%	83.3%	
Surinamese	0%	4.2%	
Antillean	1.3%	0%	
Other Western^1^	8.0%	12.5%	
Education %^b^			0.090
Low education^2^	1.3%	0%	
Medium education^2^	8.0%	29.2%	
High education^2^	86.7%	70.8%	
Smoking %^b^			0.333
Never	65.3%	58.3%	
In the past	26.7%	41.8%	
Yes	1.3%	0%	
Alcohol use %^b^			0.281
Never	82.7%	83.3%	
1 × /month or less	4.0%	12.5%	
2–4 × / month	8.0%	4.2%	
Vegan %^b^	2.7%	0%	0.513
Vegetarian %^b^	8.0%	4.2%	0.576
Mode of delivery^b^			
% cesarean	29.4%	16.7%	0.453
**Infant characteristics**			
Birthweight (gr), mean (SD)^a^	3,564.2 (487.5)	3399.7 (584.6)	0.176
Sex, % of male^b^	49.3%	75.0%	0.048

^1^Europe, North America, Oceania, Indonesia, and Japan.

^2^Based on the International Standard Classification of Education (ISCED) 2011.

• Low education (ISCED 2011): levels 0–2.

• Medium education (ISCED 2011): levels 3–4.

• High education (ISCED 2011): levels 5–8.

Statistical difference between groups tested using: ^a^Student's *t*-test, ^b^chi-square test.

CTL, control group; HS, high-stress group; SD, standard deviation; BMI, body mass index; gr, grams.

**Table 2:** Maternal energy, protein, and amino acid dietary intake.

	**Maternal dietary intake**		
**Intake component mean (SD)**	**CTL (*n* = 73)**	**HS (*n* = 21)**	***p*-value**
Energy total Kcal	2,163 (572)	1,950 (664)	0.192
Energy total Kilojoule	9,073 (2,396)	8,183 (2,780)	0.194
Total protein (gr)	77.1 (22.9)	70.7 (20.6)	0.227
Plant protein (gr)	36.2 (10.4)	30.9 (11.0)	0.059
Animal protein (gr)	41.1 (20.6)	39.9 (15.2)	0.773
**Amino acids (mg)**			
Total	74,883 (23,438)	67,295 (19,226)	0.138
Essential	33,033 (11,068)	30,133 (9,001)	0.225
Non-essential	41,850 (12,514)	37,162 (10,522)	0.093
Glutamate family	26,222 (7,599)	22,824 (6,744)	0.056
Aspartate family	18,519 (6,632)	17,125 (5,234)	0.319
Methionine	1,642 (603)	1,513 (450)	0.292
Serine family	7,684 (2,320)	7,030 (2,076)	0.224
Pyruvate family	13,382 (4,406)	12,226 (3,574)	0.224
Aromatic family	7,093 (2,298)	6,298 (1,778)	0.100
Histidine family	1,982 (659)	1,792 (577)	0.207

Food intake values are the mean of the three 24-h recall questionnaires, which reflects the overall intake of the participants during the study period.

Kcal, kilocalories; CTL, control group; HS, high-stress group; SD, standard deviation; gr, gram; mg, milligram.

All statistical differences between groups were tested using Student's *t*-test. Glutamate family members contain glutamine, arginine, and proline. Aspartate family members contain asparagine, methionine, threonine, isoleucine, and lysine. Serine family members contain serine, glycine, and cysteine. Pyruvate family members contain valine, alanine, and leucine. Aromatic family members contain phenylalanine and tyrosine. Histidine family members contain histidine.

Lifetime psychological (JTV and LSC-r questionnaire) and biological stress (hair cortisol) measurements were the same between study groups (Table 3). Perceived stress during the study period was higher in the HS group, and women in the HS groups scored higher on the PSS, EPDS, STAI-s, and STAI-t (*p* < 0.01) (Table 3). There were no differences in the HM cortisol AUCs or the HM/saliva cortisol morning peak concentrations between study groups.

**Table 3:** Maternal perceived stress scores and cortisol values between study groups.

	**CTL**	**HS**	***p*-value**
	**(*n* = 73)**	**(*n* = 24)**	
**Questionnaire-based stress scores**			
**Lifetime stress (test score)**			
JTV, median (Q1–Q3)^a^	28.0 (25.0–35.5)	29.5 (27.3–38.8)	0.459
LSC-r, median (Q1–Q3)^a^	6.0 (3–10)	5.5 (2–10)	0.432
**Perceived stress during the study period (test score)**			
PSS, mean (SD)^b^	16.52 (6.4)	20.48 (6.7)	0.001
EPDS, median (Q1–Q3)^b^	5.0 (2–7)	8.0 (5.8–11)	0.004
STAI-s, median (Q1–Q3)^b^	27.0 (23–34)	36.0 (25–40)	0.005
STAI-t, median (Q1–Q3)^b^	29.0 (26–36)	37.0 (31–40)	0.004
**Biological measures of stress: cortisol**			
**Stress over the last 3 months of pregnancy**			
Hair cortisol, median (Q1–Q3)^b^	6.0 (3.1–14.4)	7.1 (5.3–11.6)	0.280
**Stress on collection days**			
Saliva cortisol (morning peak), median (Q1–Q3)^c^	5.4 (3.4–8.0)	5.7 (3.3–8.9)	0.490
p10^b^	5.5 (3.6–8.3)	5.9 (3.0–10.5)	0.811
p17^b^	5.0 (3.3–8.0)	6.2 (4.4–8.3)	0.274
p24^b^	5.4 (4.0–7.6)	5.2 (2.1–6.1)	0.268
Human milk cortisol AUC, median (Q1–Q3)^c^	52.0 (36.9–72.1)	64.0 (41.0–91.2)	0.074
p10^b^	59.1 (41.2–81.4)	69.3 (40.7–86.4)	0.465
p17^b^	46.2 (35.3–70.9)	57.1 (44.6–93.2)	0.088
p24^b^	51.0 (36.7–70.6)	62.9 (41.0–112.8)	0.282

CTL group, control group; HS group, high-stress group; JTV, Dutch version of the Youth Trauma Questionnaire; LSC-r, life stressor checklist revised; PSS, perceived stress scale; EPDS, Edinburgh Postnatal Depression Scale; STAI-s, State and Trait Anxiety Inventory (state); STAI-t, State and Trait Anxiety Inventory (trait); Q1–Q3, 25th to 75th percentiles; SD, standard deviation; HM, human milk; AUC, area under the curve.

Statistical difference between groups tested using: ^a^Mann–Whitney *U*-test, ^b^Student's *t*-test, ^c^linear mixed effects model (correction for within-person repeated measures).

### 3.2. BAA concentrations in HM are higher in the HS group

**Figure 3:**
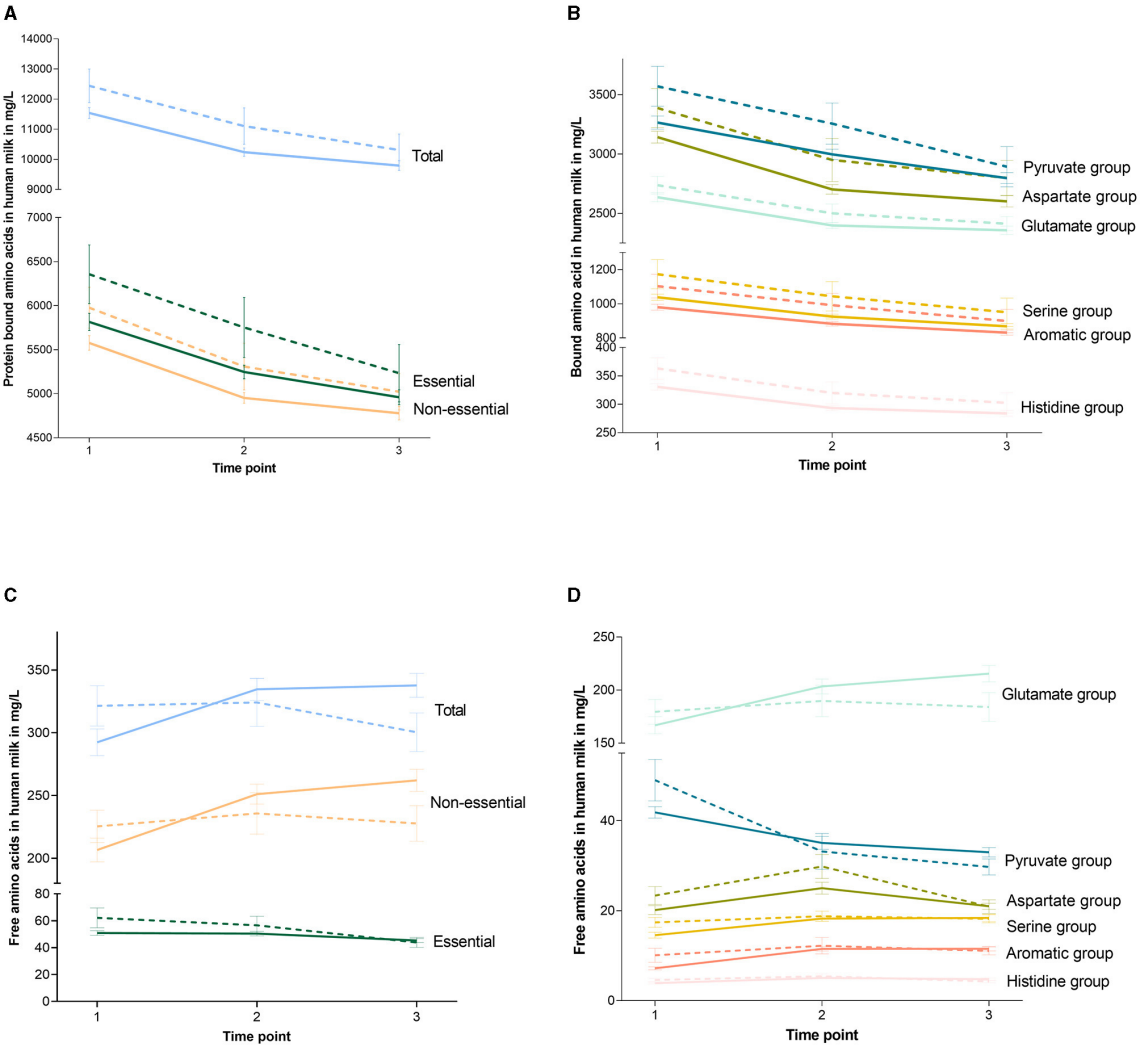
Dynamics of amino acids over the study period per study group. Graphs show the concentrations of the protein-bound amino acids **(A, B)** and the free amino acids **(C, D)** at the different collection moments. The continued line indicates the control group and the dotted line indicates the high-stress group. Error bars indicate the standard error of the mean. Glutamate family members contain glutamine, arginine, and proline. Aspartate family members contain asparagine, methionine, threonine, isoleucine, and lysine. Serine family members contain serine, glycine, and cysteine. Pyruvate family members contain valine, alanine, and leucine. Aromatic family members contain phenylalanine and tyrosine. Histidine family members contain histidine.

Table 4 shows the concentrations of BAAs in HM per study group on all collection days, and Figures 3A, B depicts the BAA dynamics over the study period per study group. Total concentrations of BAAs were higher in the milk of women in the HS group compared to the CTL group [819 (92.4, 1,547); *p* = 0.028]. This was also the case for essential BAAs [476 (55.8, 896); *p* = 0.027] and non-essential BAAs [363 (55.9, 669); *p* = 0.021] and for the concentrations of the BAA precursor groups (*p* < 0.035), except for the glutamate precursor group, which showed the same concentrations in both study groups. There were no interactions between the study group and study time point (Table 3; Figure 3). All statistical analyses were corrected for infant sex.

**Table 4:** Protein-bound amino acids in human milk over the study period per study group.

**Amino acids in μg/L, mean (SD)**	**CTL group**			**HS group**			**Difference between study groups**
	**P10 (*****n*** = **73)**	**P17 (*****n*** = **71)**	**P24 (*****n*** = **70)**	**P10 (*****n*** = **24)**	**P17 (*****n*** = **23)**	**P24 (*****n*** = **23)**	**Estimate (CI)**
Total	11,539 (1,534)	10,238 (1,158)	9,792 (1,337)	12,442 (2,739)	11,103 (2,917)	10,311 (2,547)	819 (92, 1,547)^*^
Essential	5,815 (842)	5,247 (649)	4,960 (708)	6,357 (1,632)	5,753 (1,639)	5,234 (1,564)	476 (55, 896)^*^
Non-essential	5,576 (704)	4,952 (515)	4,779 (636)	5,976 (1,134)	5,309 (1,272)	5,023 (987)	363 (55, 669)^*^
Glutamate family	2,636 (331)	2,398 (210)	2,357 (293)	2,737 (365)	2,500 (377)	2,412 (287)	92.4 (−15, 200)
Aspartate family	3,142 (432)	2,702 (337)	2,601 (397)	3,387 (806)	2,950 (872)	2,799 (707)	255 (46, 465)^*^
Methionine	205 (44.9)	180 (28.1)	163 (28.4)	213 (52.1)	200 (58.1)	164 (36.1)	7.07 (−7, 21)
Serine family	1,038 (154)	925 (121)	868 (136)	1,173 (419)	1,044 (414)	951 (399)	124 (23, 225)^*^
Pyruvate family	3,264 (473)	2,997 (373)	2,797 (389)	3,570 (826)	3,226 (835)	2,893 (811)	235 (17, 454)^*^
Aromatic family	980 (151)	883 (110)	831 (122)	1,103 (340)	992 (334)	899 (329)	110 (26, 194)^*^
Histidine family	331 (49.8)	293 (37.9)	284 (42.6)	363 (93.2)	320 (93.5)	303 (85.4)	27.6 (3, 52)^*^

Protein-bound amino acids in human milk per study group at all collection time points in μg/L.

Statistics: Group comparisons are corrected for infant sex.

^*^Significantly higher in the HS group (*p* < 0.05). No study group—time point interactions were found. Group differences and interaction were tested using linear mixed effects models to correct for within-person repeated measures.

Glutamate family members contain glutamine, arginine, and proline. Aspartate family members contain asparagine, methionine, threonine, isoleucine, and lysine. Serine family members contain serine, glycine, and cysteine. Pyruvate family members contain valine, alanine, and leucine. Aromatic family members contain phenylalanine and tyrosine. Histidine family members contain histidine.

CTL, control; HS, high stress; p, postpartum day; SD, standard deviation.

### 3.3. FAA concentrations in HM did not differ between study groups

Table 5 shows the concentrations of FAA in HM per study group on all collection days, and Figures 3C, D depicts the FAA dynamics over the study period per study group. Total concentrations of FAA over the entire study group did not differ between the HS and CTL groups, nor did concentrations of essential FAA, non-essential FAA, and the concentrations of the FAA precursor family groups. All statistical analyses were corrected for infant sex.

**Table 5:** Free amino acids in human milk over the study period per study group.

**Amino acid in μg/L, mean (SD)**	**CTL group**			**HS group**			**Difference between study groups**
	**P10 (*****n*** = **73)**	**P17 (*****n*** = **71)**	**P24 (*****n*** = **70)**	**P10 (*****n*** = **24)**	**P17 (*****n*** = **23)**	**P24 (*****n*** = **23)**	**Estimate (CI)**
Total	292 (91.2)	334 (74.3)	338 (79.2)	321 (78.8)	324 (91.4)	300 (73.8)	−16.1 (−48.4, 16.3)^a^
Essential	50.8 (15.4)	50.4 (13.9)	45.3 (12.9)	62.2 (36.1)	56.6 (32.8)	43.8 (17.4)	2.1 (−4.0, 8.3)^a^
Non-essential	207 (80.5)	251 (66.5)	262 (73.1)	225 (62.9)	235 (79.0)	227 (68.1)	−16.5 (−46.0, 13.1)^a^
Glutamate family	167 (68.8)	203 (59.0)	215 (64.9)	179 (57.1)	190 (71.5)	184 (64.9)	−13.8 (−40.2, 12.6)^a^
Aspartate family	20.1 (8.5)	25.0 (11.2)	21.0 (5.7)	23.4 (9.8)	29.8 (12.9)	20.9 (7.2)	1.6 (−1.1, 4.3)
Methionine	1.1 (0.4)	4.1 (5.5)	1.2 (0.8)	1.5 (1.3)	6.6 (7.9)	1.5 (2.1)	0.5 (0.2, 0.8)^*^
Serine family	14.6 (5.6)	18.3 (4.9)	18.4 (7.2)	17.4 (5.4)	18.8 (5.4)	18.2 (4.3)	1.0 (−1.0, 3.0)
Pyruvate family	41.8 (11.0)	35.1 (12.1)	33.0 (8.9)	49.0 (22.7)	33.2 (19.1)	29.7 (8.5)	−2.2 (−6.3, 1.9)^a^
Aromatic family	7.2 (2.8)	11.5 (4.0)	11.5 (4.0)	10.1 (7.7)	12.2 (8.7)	11.1 (4.2)	0.9 (−0.7, 2.5)^a^
Histidine family	3.9 (1.7)	5.1 (1.3)	4.8 (2.4)	4.6 (1.7)	5.4 (2.6)	4.3 (1.4)	0.2 (−0.4, 0.9)

Free amino acids in human milk per study group at all collection time points in μg/L.

^*^Significantly higher in the HS group (*p* < 0.05). Group comparisons are corrected for infant sex.

^a^Significant study group—time point interaction (*p* < 0.05).

Statistics: Group differences and interactions were tested using linear mixed effects models to correct for within-person repeated measures.

Glutamate family members contain glutamine, arginine, and proline. Aspartate family members contain asparagine, methionine, threonine, isoleucine, and lysine. Serine family members contain serine, glycine, and cysteine. Pyruvate family members contain valine, alanine, and leucine. Aromatic family members contain phenylalanine and tyrosine. Histidine family members contain histidine.

CTL, control; HS, high stress; p, postpartum day; SD, standard deviation.

### 3.4. Bound methionine does not differ between study groups, free methionine is higher in the HS group

Despite higher concentrations of BAA of the aspartate precursor family in the HS group [255 (46.0, 465); *p* = 0.017], to which protein-bound methionine belongs, protein-bound methionine concentrations did not differ between the HS and CTL groups. In contrast, free methionine concentrations were higher in the HS group compared to the control group [0.49 (0.21, 0.76); *p* = 0.001]. All statistical analyses were corrected for infant sex.

### 3.5. Dynamics of FAAs in HM over the first month of lactation differ between study groups

In general, the BAA concentrations in HM decreased over the study period (*p* < 0.001), while the FAA concentrations increased (*p* = 0.019). As indicated in Table 5, for total FAA, there was an interaction between the study group and study time point. This was the same for essential and non-essential FAAs, as well as glutamate, pyruvate, and aromatic precursor groups. The different dynamics over the study period between the HS and CTL groups are depicted in Figures 3C, D. All statistical analyses were corrected for infant sex.

### 3.6. HM BAAs are positively associated with HM cortisol levels

Total BAA concentrations, as well as essential BAAs and non-essential BAAs, were positively associated with the HM cortisol AUC [5.54 (0.73, 10.35); *p* = 0.024, 2.66 (0.01, 5.30); *p* = 0.049 and 3.07 (0.85, 5.29); *p* = 0.007, respectively]. Protein-bound methionine was not associated with the HM cortisol AUC. Total FAA, essential FAA, non-essential FAA, and free methionine were also not associated with the HM cortisol AUC, at the separate study time points.

## 4. Discussion

In this study, we explored associations between maternal stress and the AA composition of HM. We demonstrated that perceived and biological maternal stresses in the first month postpartum were positively associated with the concentrations of BAAs in HM. In particular, BAA concentrations were increased in the HM of mothers with high-stress levels, with the exception of bound methionine. Methionine was, contrary to our hypothesis, not associated with maternal stress. However, while the overall concentrations of FAAs in HM did not differ between study groups, free methionine was higher in the HM of mothers with high levels of perceived stress.

Our results are in line with previous animal experimental studies (22, 23). In stressed dams, an increase in some of the milk BAAs was observed, while milk FAAs were overall not affected by stress exposure. To date, human studies on the effect of maternal stress on the AA composition of HM are scarce. One study investigated the influence of maternal postpartum stress on the metabolome of HM, including three AAs, and found that high-stress levels were positively associated with the concentrations of tryptophan and tyrosine. However, after correction for multiple testing, these associations disappeared (16). Another study by Ziomkiewicz et al. found that proteins in HM were not associated with maternal psychological or biological stress (15). This difference may be attributed to the fact that Ziomkiewicz et al. measured whole proteins instead of specific AAs.

The biological mechanism behind the observed associations between maternal stress and HM AA composition is not yet clear. In fact, the process behind the transport of AAs from the maternal bloodstream into the mammary gland and into the HM is complex and not yet fully understood (21). Both BAAs and FAAs in HM can be transported from the maternal circulation, and BAAs can be synthesized out of FAAs by the mammary gland itself (21). It has further been demonstrated, in contrast to our current results in HM, that almost all AAs in the maternal plasma are decreased as a result of maternal stress (18–20). The origin of such a reduction is not fully elucidated. It might be due to the influence of catecholamines produced during stress, which exert an anti-insulin effect on the metabolism of AAs (18–20). As we observed higher concentrations of HM BAAs in the HS group but no association between stress and FAAs in HM, this may indicate an increased active transport of AAs from the maternal circulation into HM and/or an increased synthesis of BAAs in the mammary gland.

Exactly how and to what extent stress influences transport processes also remains to be elucidated. As previous research showed associations between glucocorticoid levels and the expression of certain AA transporters in the mammary gland, e.g., the system L and the system y^+^ amino acid transporters, one could hypothesize that cortisol is involved in the mechanisms leading to these stress-induced changes in HM (21, 48). Indeed, we observed a positive association between cortisol levels and BAA levels in HM, which was also found in previous animal experimental research (22). A possible explanation for the fact that cortisol values were not elevated in the HS group but were positively associated with HM BAAs might be that the sample size of the HS group was not sufficient to detect a statistically significant difference. In fact, there was a “trend” toward a higher HM cortisol AUC in the HS group (*p* = 0.074).

We further focused specifically on methionine, as in mice, maternal stress has led to reduced methionine in the brain and plasma of the offspring, which was associated with later-life cognitive deficits (24). Contrary to our hypothesis, bound methionine levels were not decreased in the HM samples in our HS group, and free methionine levels were even higher in mothers with high perceived stress levels. This was an unexpected result, but it can be speculated that the absence of an increase in protein-bound methionine is the result of stress-induced breakdown into free methionine, which would result in an increase in the free form of this essential AA. In addition, another possible explanation for the absence of an increase in protein-bound methionine is that while some transporters of AAs in the mammary gland seem to be upregulated under the influence of cortisol, one of the three transporters that facilitate the transport of methionine into HM is rather downregulated by glucocorticoids (21, 48). Unfortunately, the influence of cortisol on the other two methionine transporters remains so far unknown (21).

Increased concentrations of BAA in the HM of mothers in the HS group may be due to the fact that mothers with high levels of stress produce less milk, in general, compared to mothers with lower levels of stress (49–51). When AA transport into milk is maintained at the same level, the concentrations of BAA in HM will then increase. This might lead to the appropriate transmission of these important nutrients to the infant. On the other hand, this would also mean that the infant might be at risk of receiving inappropriate amounts of methionine, which was not increased in HM under stressful circumstances, as was shown in our results. As we did not measure the total milk volume production during the day or AA concentrations in the infant, this remains speculation and awaits future research.

During lactation, milk-specific proteases break down HM proteins into FAAs. HM further contains protease activators and protease inhibitors (52). Over the first month of lactation, protease inhibitors decrease, which leads to an increase in total FAAs, but subsequently, this also contributes to a decrease in HM BAAs over time (52). Indeed, a decrease in BAA and an increase in total FAA over time were observed for the mothers in our study, except for the concentrations of HM FAAs of the mothers in the HS group, where the total FAA levels decreased over time. The different dynamics of FAA observed in the HS group might be due to a different regulation or production of proteases under the influence of stress. Another explanation for the decrease in total HM FAA under stressful circumstances is that FAAs are more often bound in response to stress, resulting in the increase in HM BAA observed in the HS group.

The strengths of our current study are its longitudinal design and the timing and frequency of HM sample collection. The first month postpartum is a sensitive time window that has been frequently missed in earlier HM research, but since breastfed infants depend on HM during this period as their only source of nutrition these first weeks after birth represent a very important period. Furthermore, the fact that mothers were exposed to a stressor (i.e., infant hospitalization) ensured that the study population contained participants with established high levels of perceived stress. Additionally, extensive information on both maternal psychological and biological stress was collected, and the HS and CTL groups were similar at baseline with regard to lifetime stress levels or other important characteristics that could have influenced the relationship between stress and HM AA levels. The limitations of this study are, first, the relatively small sample size, especially in the HS group. Second, current perceived stress scores were only measured once, i.e., at the end of the study. Therefore, we were not able to investigate whether the different dynamics of FAA in the HS group were related to changes in the amount of stress experienced at that exact moment. Moreover, because of the exploratory nature of this study, we decided not to correct for multiple testing, even though we did perform a relatively large number of statistical tests, which increases the likelihood of a type 1 error. Therefore, our results should be interpreted with caution, and future studies should demonstrate whether these findings can be replicated. In addition, no reliable information was collected about infant feeding modes and additional feeding practices. The relatively high loss to follow-up and the fact that this was higher in the HS group compared to the CTL group (19 vs. 23%, respectively) may have caused selection bias. Dropout of women who may have been more stressed, especially in the HS group, may have led to an underestimation of associations between maternal stress and AA. Finally, selection bias might have contributed to the fact that our cohort mostly consisted of healthy and highly educated women. Furthermore, the majority of our participants were of Western ethnic background. This may limit the generalizability of the results.

The results of our study suggest that mothers with high-stress levels have increased concentrations of BAA in their milk. The increased concentrations of BAA that we found may partially compensate for the higher nutritional requirements of infants under stressful circumstances. However, as impaired growth and development have been observed in children of mothers with high amounts of stress in the postnatal period, this increase in AA in the HM might not be sufficient to maintain optimal growth and development. Moreover, the increase was not observed for protein-bound methionine or total HM FAA.

In conclusion, findings from this unique prospective cohort study suggest that there is a relationship between maternal stress in the first month postpartum and the AA concentrations of HM during this time. Our findings emphasize the importance of the maternal psychological state during lactation. In the last few years, attention to the prevention of the detrimental consequences of stressful experiences in early life has increased. Stress reduction programs for children and parents have been developed, such as family-integrated care and relaxation therapy using relaxing music (53–59), with one study demonstrating effects on milk composition (59). Future research should replicate our findings, investigate the entire period of lactation, and focus on to what extent these stress-induced changes in human milk composition are of clinical importance for short- and long-term infant development and health.

## Data availability statement

The raw data supporting the conclusions of this article will be made available by the authors, without undue reservation.

## Ethics statement

The studies involving human participants were reviewed and approved by Ethics Committee of the Amsterdam University Medical Centre, AMC. The patients/participants provided their written informed consent to participate in this study.

## Author contributions

EN, JG, SR, LS, and AK designed the research. HJ and EN conducted the research. LS, JG, and AK provided essential materials. HJ, JH, and SR analyzed the data or performed statistical analysis. JH, HJ, BK, SR, and AK accessed, verified, and interpreted the data. HJ, SR, and AK wrote the first version of the manuscript. AK had primary responsibility for the final content. All authors critically read and contributed to finalizing the manuscript, had full access to all the data in the study, and accept responsibility to submit it for publication.
